# Multialloy Au‐Co‐Pd Nanopillars‐in‐Oxide Hybrid Metamaterials with Tunable Optical and Magnetic Properties

**DOI:** 10.1002/smsc.202500455

**Published:** 2025-11-10

**Authors:** Vasundhara Acharya, Juanjuan Lu, Jiawei Song, Ping Lu, Alessandro R. Mazza, Jianan Shen, Zihao He, Juncheng Liu, Hongyi Dou, Yizhi Zhang, Zhongxia Shang, Aiping Chen, Haiyan Wang, Di Zhang

**Affiliations:** ^1^ Department of Materials Science and Engineering University of Texas at Arlington Arlington TX 76019 USA; ^2^ School of Materials Engineering Purdue University West Lafayette IN 47907 USA; ^3^ Sandia National Laboratories Albuquerque NM 87185 USA; ^4^ Center for Integrated Nanotechnologies Los Alamos National Laboratory Los Alamos NM 87545 USA; ^5^ Materials Science and Technology Division Los Alamos National Laboratory Los Alamos NM 87545 USA

**Keywords:** anisotropy, optical properties, self‐assembled, surface energy, vertically aligned nanocomposite

## Abstract

Vertically aligned nanocomposite (VAN) thin films have attracted extensive research interests in recent years owing to their unique structure anisotropy and broad integration compatibility with versatile material systems, which open enormous possibilities in the applications of electronic and photonic devices. In this article, to further expand the materials selection in oxide‐metal alloys VAN structure, self‐assembled BaTiO_3_ (BTO): (Au*‐*Co‐Pd) and BTO: (Au‐Pd) nanocomposite films are integrated using a simplified oxide‐metal strips deposition method via pulsed laser deposition (PLD). Microstructural characterization results confirm the epitaxial film quality and vertically grown Au*‐*Co‐Pd and Au‐Pd alloyed nanopillars in both nanocomposite films, where the elemental segregation of Au, Co, and Pd is primarily due to the differences in their surface energies. Both experimental and simulated optical data show the highly tailorable optical properties of the hybrid films such as localized surface plasmon resonance and hyperbolic dispersion wavelength shifts in the visible to near‐infrared wavelength region. The successful integration of multiple metal elements via the one‐step oxide‐metal strips method in PLD demonstrates the wide feasibility of integrating diverse materials systems into VAN structure toward multifunctional property coupling for electronic, photonic, and energy devices applications.

## Graphical abstract

Self‐assembled vertically aligned nanocomposite (VAN) BTO: (Au*‐*Co‐Pd) and BTO: (Au‐Pd) VAN thin films are grown using a simplified oxide‐metal strips deposition method in pulsed laser deposition. The hybrid VAN films exhibit tunable localized plasmonic surface resonance and hyperbolic dispersion characteristics in the visible to near‐infrared wavelength region.

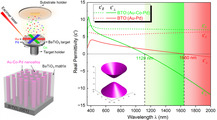

## 1 Introduction

Fabrication of thin film heterostructures and superlattices has been an effective approach to achieve exotic physical properties such as enhanced ferroelectricity,^[^ ^1^, ^2^ ^]^ superconductivity,^[^ ^3^, ^4^ ^]^ and complex topologies.^[^ ^5^, ^6^ ^]^ Vertically aligned nanocomposites (VANs) are a novel nanostructured architecture in which two immiscible phases self‐assemble during film growth.^[^ ^7^, ^8^, ^9^, ^10^ ^]^ The VAN morphology enables the combination of distinct properties from each constituent of material, facilitating enhanced charge transfer and strain engineering at the heterointerfaces and giving rise to exceptional properties such as ferroelectric, ferromagnetic, multiferroic, plasmonic, and superconducting characteristics.^[^ ^11^, ^12^, ^13^, ^14^, ^15^, ^16^, ^17^, ^18^ ^]^ Beyond the first explored oxide‐oxide VAN thin films, in the past few years, the materials combination of VANs has been extended to oxide‐metal,^[^ ^19^, ^20^, ^21^, ^22^, ^23^ ^]^ nitride‐metal,^[^ ^24^, ^25^, ^26^, ^27^ ^]^ and oxide‐nitride^[^ ^28^, ^29^, ^30^ ^]^ systems, paving the avenue of diverse materials integration for multifunctional device applications.

It is found that some noble metals (such as Au^[^ ^20^, ^21^, ^22^, ^23^ ^]^ and Cu^[^ ^31^ ^]^) and ferromagnetic metals (such as Co,^[^ ^32^, ^33^ ^]^ Ni,^[^ ^19^, ^34^ ^]^ Fe^[^ ^35^ ^]^) can be easily incorporated with oxide (nitride) phases to form oxide (nitride)‐metal VAN structured films. On the contrary, it is challenging to integrate many other transition metal elements (such as Ag, Pt, Ir, and so on) to form oxide‐ or nitride‐based VAN films. This discrepancy, though not fully explored yet, can be attributed to many thermodynamic and kinetic parameters during film growth such as material crystallographic symmetry, lattice mismatch, surface energy, wettability, temperature, and oxygen partial pressure.^[^ ^10^ ^]^ One feasible way is to integrate other metallic elements into the existing oxide (nitride)‐metal structures to form multiphase VAN systems. For instance, by growing oxide‐Au VAN as seed layers at the bottom followed by the growth of the second oxide‐Ag layers on the top, the deposited film structures become oxide‐Au_*x*_Ag_1‐*x*_ alloyed VAN films.^[^ ^36^, ^37^ ^]^ To further discover the great potential of this method of incorporating immiscible species in a VAN framework, various multiphase VAN systems have been explored recently such as all‐oxide systems,^[^ ^38^, ^39^ ^]^ oxide‐metal alloys,^[^ ^36^, ^37^, ^40^, ^41^, ^42^, ^43^ ^]^ nitride‐metal alloys,^[^ ^29^, ^44^ ^]^ oxide‐oxide‐metal,^[^ ^23^, ^45^ ^]^ and oxide‐nitride‐metal^[^ ^46^ ^]^ systems. The multiphase VAN thin films exhibit some intriguing properties such as broader plasmonic resonance tuning range^[^ ^36^, ^37^, ^41^ ^]^ and strong magneto‐optic Kerr effect^[^ ^29^, ^41^, ^44^, ^46^ ^]^ that are usually difficult to obtain in single phase thin films. In another example, the room‐temperature self‐biased magnetoelectric switching effect is achieved in the Na_0.5_Bi_0.5_TiO_3_ (NBT)‐NiO‐NiFe_2_O_4_ (NFO) three‐phase VAN film, where NBT forms the film matrix with vertical NiO‐coated NFO nanocolumns embedded in it.^[^ ^39^ ^]^ The multiple‐phase nature in those multiphase VAN systems allows for the exploitation of distinct characteristics from each material, resulting in many enhanced and novel functionalities that surpass those of the single‐phase systems. Moreover, with the increased complexity of VAN structures, combining high throughput synthesis and machine learning could expedite the discovery of new functionalities and new systems.^[^ ^47^, ^48^, ^49^ ^]^

**Figure 1:**
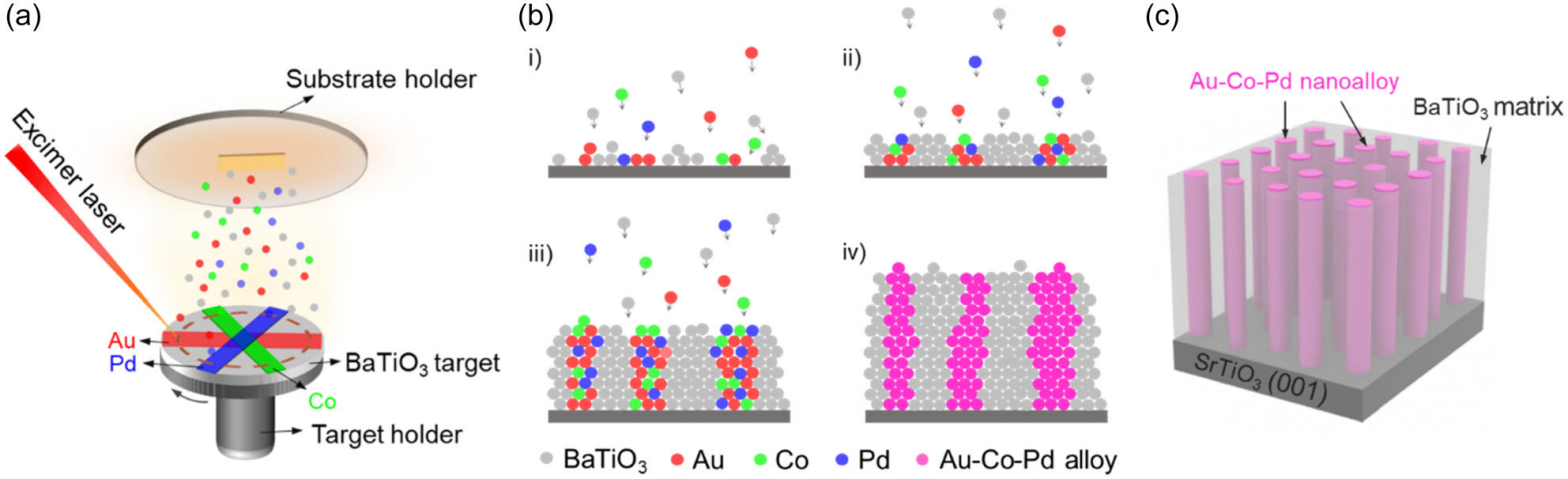
a) Schematic illustrations of the oxide‐metal strips deposition process of BTO: (Au‐Co‐Pd) nanocomposite film. b) Schematic of the different stages i–iv) of the growth of BTO: (Au‐Co‐Pd) composite film. c) 3D schematic of the as‐deposited BTO: (Au‐Co‐Pd) thin film.

Palladium (Pd) is a highly valuable noble metal with exceptional catalytic properties. Owing to its high surface reactivity with reactants (such as H_2_, CO, or alkenes) and d‐band electron configuration, Pd becomes an excellent catalyst for hydrogeneration, dehydrogenation, and C—C coupling reactions.^[^ ^50^, ^51^ ^]^ The excellent catalytic properties, resistant to oxidation and corrosion, and relatively lower cost (compared to platinum [Pt]) enable Pd to have wide applications in fuel cells, hydrogen storage, and automotive technologies. Therefore, to integrate Pd element into VAN, film systems is meaningful as it can open potential possibilities of VAN films in catalysts and energy storage fields. In this work, we grow a BaTiO_3_ (BTO): (Au*‐*Co‐Pd) nanocomposite film using pulsed laser deposition (PLD) to achieve highly tunable and controllable physical properties. Different from the previous synthesis approach of multiphase VAN systems where the nanocomposite targets with predetermined composition ratios were made using the conventional solid‐state mixing and sintering method, here we simply glued Au, Co, and Pd metal strips onto a pure BTO target by silver paste (see **Figure** **1**a). As the schematic shows, a high energy laser beam strikes on the rotating target and ablates material from BTO target pellet as well as Au, Co, and Pd metal strips. As the evaporated atoms arrive the SrTiO_3_ (STO) substrate surface, the nanocomposite film starts to grow. **Table** **1** summarizes the crystal structures, lattice parameters (*a*), and surface energies (*E*_surf_) of different materials in this work.^[^ ^36^, ^41^, ^52^, ^53^ ^]^ As we can see that Pd has the same crystal structure and similar *a* and *E*_surf_ values with Au, indicating its good lattice matching and wettability with Au. Figure 1bi–iv shows the different growth stages of the BTO: (Au‐Co‐Pd) nanocomposite film. Because of the close lattice parameters and surface energy values between Au, Pd, and Co, all the metal atoms tend to mix and form Au‐Co‐Pd alloyed nanopillars in the BTO matrix, as illustrated in Figure 1c. For comparison, the other BTO: (Au‐Pd) film is deposited by the same oxide‐metal strips deposition approach. The microstructures of the as‐deposited nanocomposite films are comprehensively characterized by X‐ray diffraction (XRD) and transmission electron microscopy (TEM). The physical properties including localized surface plasmonic resonance (LSPR), dielectric permittivity, and magnetism for both BTO: (Au*‐*Co‐Pd) and BTO: (Au‐Pd) films are investigated as well. The successful synthesis of the BTO: (Au*‐*Co‐Pd) and BTO: (Au‐Pd) VAN thin films highlight the feasibility of growing versatile oxide (nitride)‐metal alloys VAN structures using simplified oxide‐metal strips deposition method in PLD.

**Table 1:** Summary of the crystal structure, lattice parameter, and surface energy of the materials in this study.

Material	Crystal structure	Lattice parameter [Å]	*E*_surf_ [J m^−2^]
Au	FCC	4.072^[^ ^41^ ^]^	1.55^[^ ^36^ ^]^
Pd	FCC	3.890^[^ ^52^ ^]^	2.05^[^ ^53^ ^]^
Co	HCP	3.544^[^ ^41^ ^]^	2.55^[^ ^53^ ^]^
BaTiO_3_	Perovskite	4.031^[^ ^41^ ^]^	1.24^[^ ^41^ ^]^
SrTiO_3_	Perovskite	3.905^[^ ^41^ ^]^	1.26^[^ ^41^ ^]^

## 2 Results and Discussions

**Figure 2:**
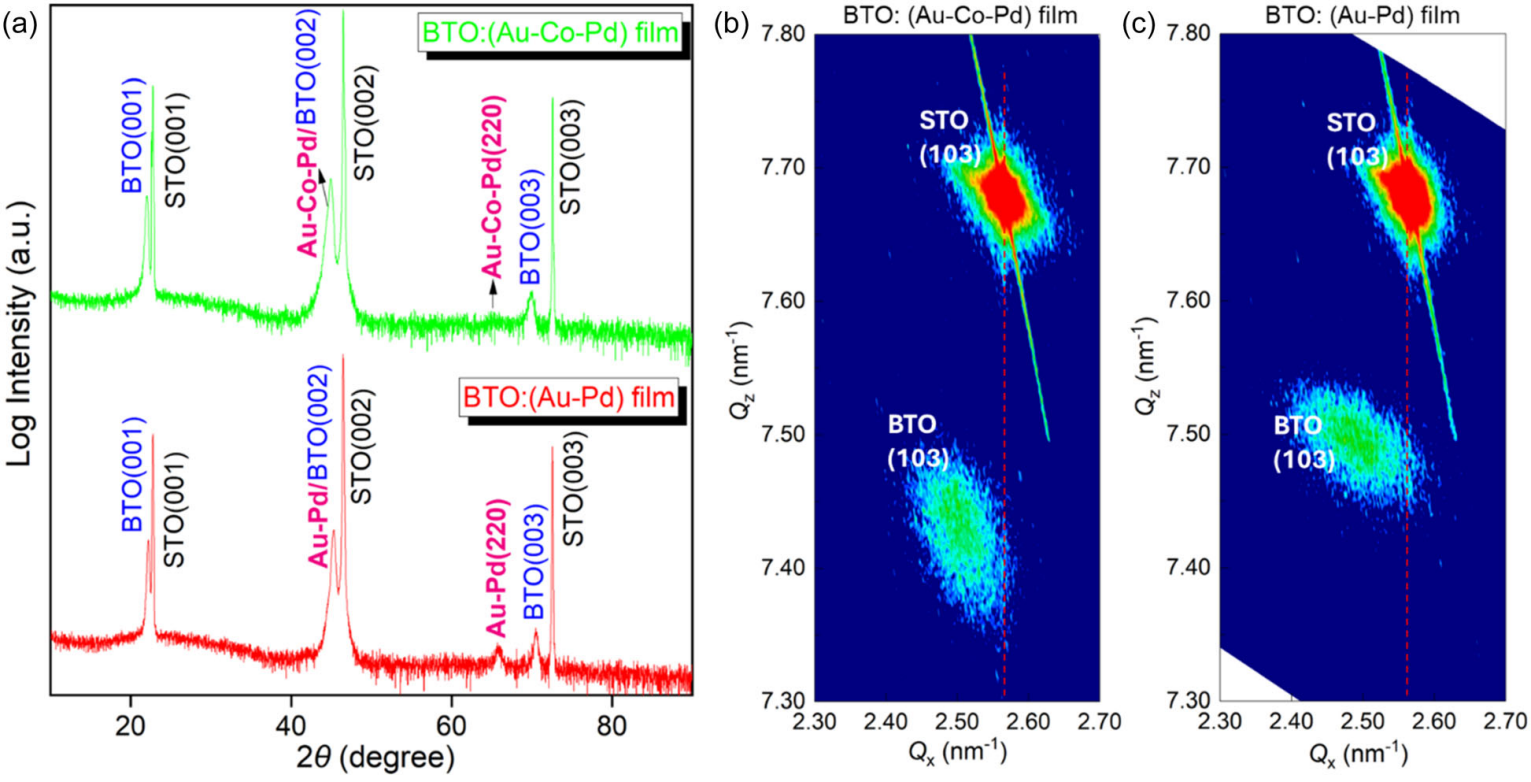
a) The XRD *θ*‐2*θ* intensities of BTO: (Au*‐*Co‐Pd) and BTO: (Au‐Pd) films grown on STO (001). RSMs around (103) Bragg's reflections of b) BTO: (Au*‐*Co‐Pd) and c) BTO: (Au‐Pd) nanocomposite films.

The phases and crystallinity of the as‐deposited nanocomposite thin films were first investigated by XRD. The XRD *θ*‐2*θ* intensities of both the BTO:(Au*‐*Co‐Pd) and BTO:(Au‐Pd) films are plotted in **Figure** **2**a. The BTO phase in both films grew epitaxially along (00l) orientation on the STO (001) substrate due to a small lattice mismatch between BTO (*a* = 4.031 Å) and STO (*a* = 3.905 Å). In contrast, the alloyed phases (i.e., Au*‐*Co‐Pd and Au‐Pd) show both (002) and (220) peaks, indicating two primary textured growths. The reciprocal space mapping (RSM) measurements were performed around (103) Bragg's reflection of the film and substrate. Figure 2b,c shows that both BTO: (Au*‐*Co‐Pd) and BTO: (Au‐Pd) films are relaxed from the STO (001) substrate, as indicated by the lack of vertical alignment of the (103) film with respect to the STO (103) peak. This strain relaxation can be attributed to the film and substrate lattice mismatch as well as the incorporation of secondary metal phases that led to the local lattice distortion and poorer film quality.^[^ ^54^, ^55^, ^56^ ^]^

**Figure 3:**
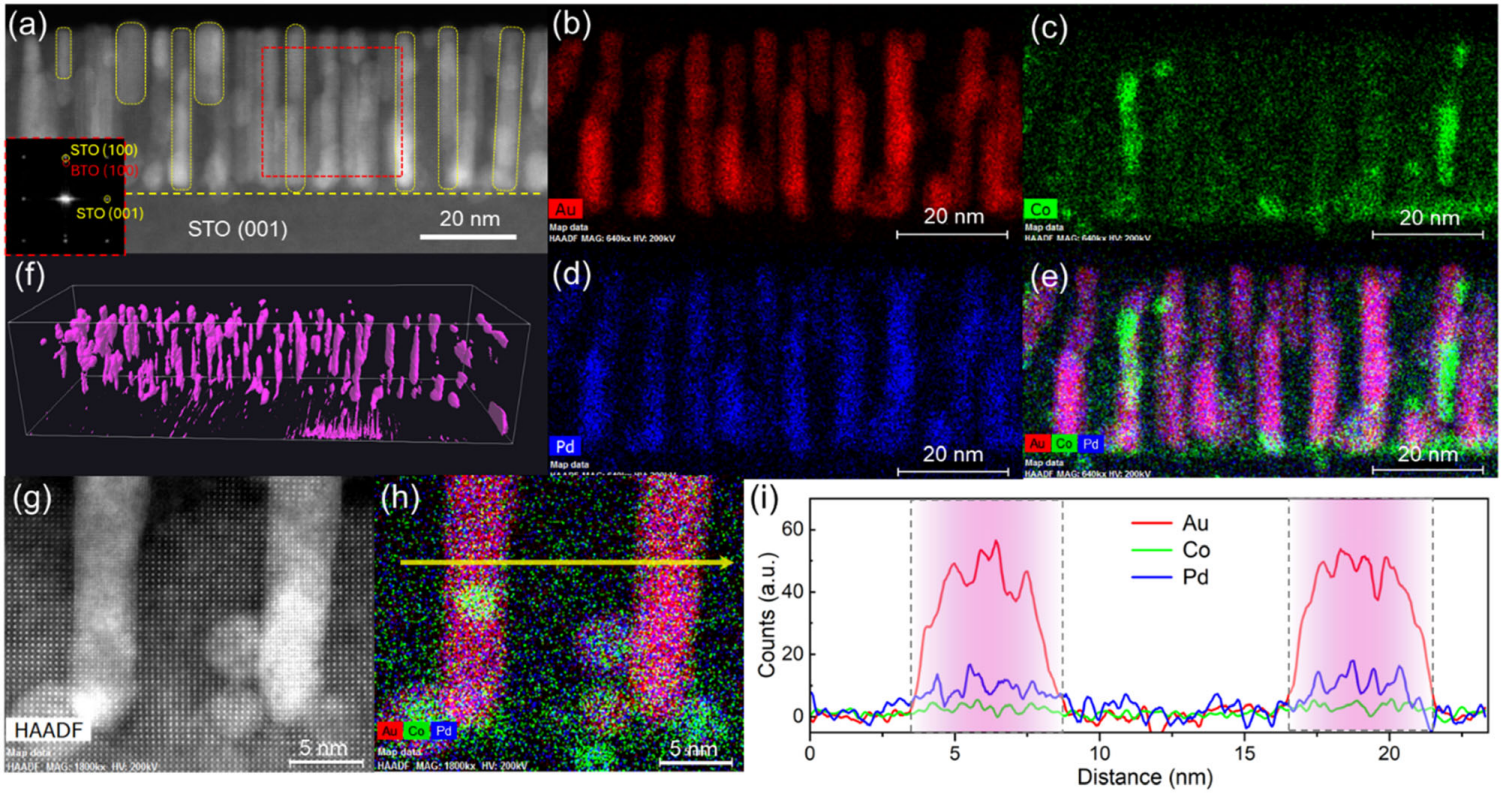
a) Low‐magnification cross‐sectional STEM image of the BTO: (Au*‐*Co‐Pd) composite film. The inset is the FFT image extracted from the red dashed square region in (a). EDS elemental mappings of b) Au, c) Co, d) Pd, and e) composite maps. f) 3D electron tomography showing the distribution of the Au*‐*Co‐Pd alloyed nanopillars in the film. g) HRSTEM image with corresponding h) EDS map and i) line profile showing the elemental intensity of Au, Co, and Pd across the alloyed nanopillars (denoted by the yellow arrow in (h).

The microstructures of the BTO: (Au*‐*Co‐Pd) and BTO: (Au‐Pd) nanocomposite films were further characterized by scanning TEM (STEM) and energy dispersive X‐ray spectroscopy (EDS). **Figure** **3**a shows the cross‐sectional STEM image of the BTO: (Au*‐*Co‐Pd) film. As expected, the metallic nanopillars grow vertically throughout the film to form the VAN morphology. The Fast Fourier transform (FFT) image (inset in Figure 3a) identifies the BTO(001)//STO(001) matching relation, indicating a cube‐on‐cube epitaxy growth. The EDS elemental of Au, Co, Pd, and composite maps shown in Figure 3b–e clearly show the elemental distribution within the metallic nanopillars. It is seen that Au and Pd are distributed quite uniformly throughout those pillar regions, while Co shows an elemental segregation and is only rich in certain regions within several nanopillars. The elemental segregation is primarily attributed to the different surface energy (*E*_surf_) values of metals (Table 1). As *E*_surf(Au)_ < *E*_surf(Pd)_ < *E*_surf(Co)_, the Au atoms have a better wettability with STO substrate and BTO matrix, thus becoming a primary element of forming the Au‐Pd‐Co alloyed nanopillars; in contrast, due to the higher *E*_surf_ of Co, Co atoms tend to be agglomerated and enriched in the particular regions of nanopillars. Figure 3f presents a snapshot from the 3D electron tomography experiment (see Movie S1, Supporting Information) to visualize the 3D spatial distribution of the Au*‐*Co‐Pd alloyed nanopillars. A high‐resolution STEM (HRSTEM) image shown in Figure 3g shows no large lattice distortion at the BTO/(Au*‐*Co‐Pd) interface, and the composite EDS map in Figure 3h confirms the uniform distribution of Au and Pd and segregation of Co in the nanopillars. To further compare the composition ratio of different metal elements, a line scan profile is retrieved and plotted in Figure 3i. It is clear that Au occupies the highest concentration (>50%) in the alloyed pillars; In contrast, Pd shows the concentration ≈10%–15%, slightly higher than that of Co (<5%). Figure S1 and S2, Supporting Information, shows the STEM images and EDS results of different regions in the BTO: (Au*‐*Co‐Pd) and BTO: (Au‐Pd) VAN films, where the similar elemental composition ratios are observed. As discussed before, the different intensity ratio in Au*‐*Co‐Pd alloyed nanopillars is mainly attributed to their different surface energy values and wettability with the BTO matrix and STO substrate. Furthermore, since the growth of the VAN thin films is a very dynamical process, by tuning the growth parameters such as laser ablation energy, growth temperature, and oxygen partial pressure, we should able to further change the film growth morphology and elemental distribution in the VAN structures and obtain some interesting alloyed nanopillar structures, such as core–shell,^[^ ^41^, ^43^, ^44^, ^46^ ^]^ “nanosandwich”,^[^ ^43^ ^]^ and “nanodomino”.^[^ ^36^ ^]^

**Figure 4:**
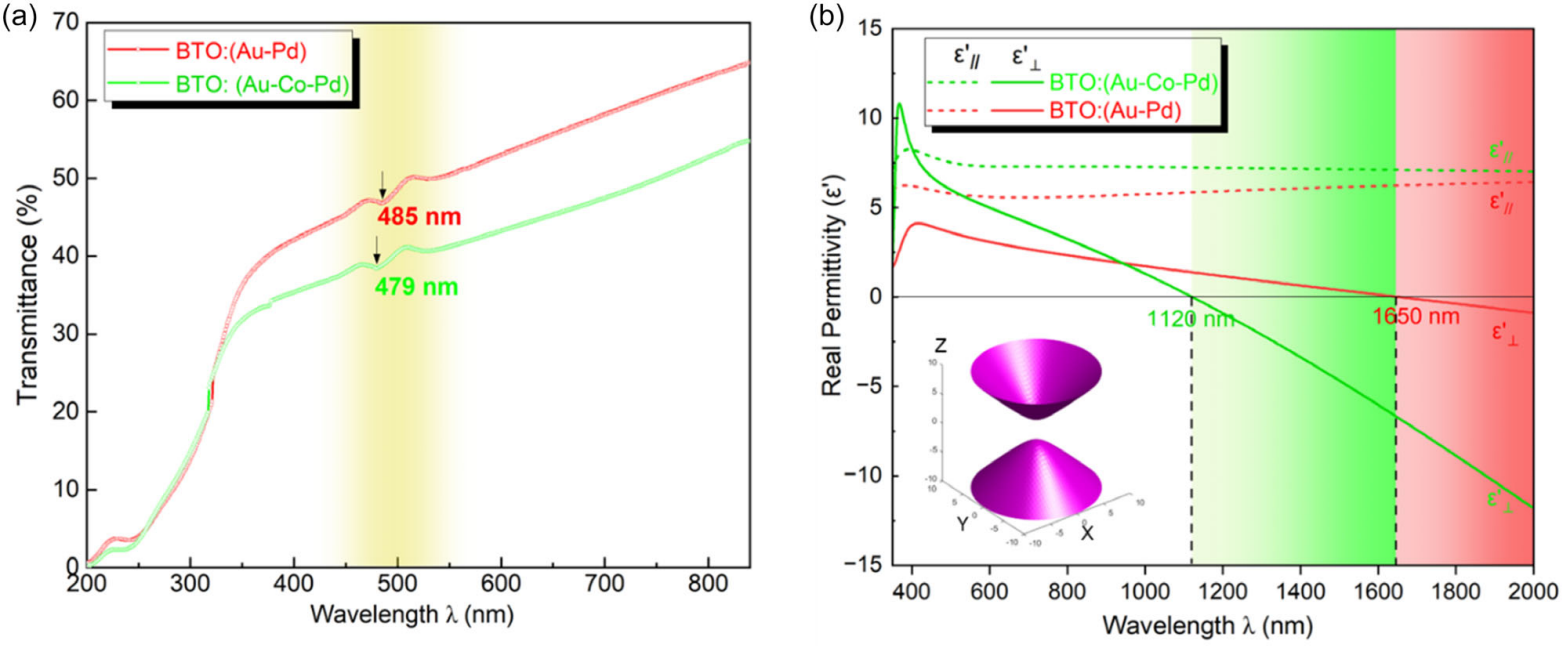
a) Transmittance spectra of the BTO: (Au‐Pd) and BTO: (Au*‐*Co‐Pd) thin films. b) The real of the IP (*ε*′_∥_) and OOP (*ε*′_⊥_) permittivity components of the BTO: (Au‐Pd) and BTO: (Au*‐*Co‐Pd) thin films, with inset showing the iso‐frequency surface topology at the hyperbolic dispersion wavelength regime.

LSPR arises when surface electrons at a metal–dielectric interface oscillate in resonance with incident light. This resonance wavelength can be tuned by modifying the metal type, surface geometry, or surrounding dielectric environment, allowing for tailored light‐matter interactions at the nanoscale.^[^ ^57^ ^]^ The optical properties of the two as‐deposited VAN thin films were then investigated by UV–Vis spectroscopy. **Figure** **4**a shows the transmittance (T%) spectra of the BTO: (Au‐Pd) and BTO: (Au*‐*Co‐Pd) hybrid films. For BTO: (Au‐Pd) film, a distinct LSPR valley is identified at 485 nm. In contrast, the BTO: (Au*‐*Co‐Pd) hybrid film shows less T% intensity with the LSPR valley at 479 nm. Previous studies show that the LSPR position performs a blue shift by adding Ag into the original Au phase to form Au_0.4_Ag_0_._6_ VAN film.^[^ ^36^ ^]^ Similarly, the addition of Pd and Co to Au leads to the LSPR shift from ≈580 nm (pure Au)^[^ ^21^ ^]^ to ≈480 nm (Au*‐*Co‐Pd) in this work. Next, the finite element analysis was conducted in COMSOL Multiphysics to simulate the LSPR in BTO: Au, BTO: (Au‐Co), BTO: (Au‐Pd), and BTO: (Au*‐*Co‐Pd) hybrid VAN structures. The refractive index (n, k) values of nanoalloys were estimated using Maxwell–Garnett (MG) and Bruggeman models based on effective medium approximation (EMA) theory,^[^ ^58^, ^59^ ^]^ as plotted in Figure S3, Supporting Information. Due to the small fraction and nonuniform distribution of Co and Pd in the alloyed nanopillars, the *n* and *k* values derived from MG model were selected for the COMSOL simulation. Figure S4, Supporting Information, shows the electric field maps (EFMs) of the BTO: Au, BTO: (Au_0.9_Co_0.1_), BTO: (Au_0.8_Co_0.05_Pd_0.15_), and BTO: (Au_0.7_Pd_0.3_) hybrid thin films at different normal incident light wavelengths. Figure S4a, Supporting Information, shows that the highest SPR intensity of BTO‐Au occurs at around 600 nm. By including Co and Pd to form Au‐Co, Au‐Pd, and Au*‐*Co‐Pd multialloys, the LSPR peak intensity in those hybrid systems show broadening and noticeable blue‐shifts compared to that of BTO: Au. From S4b to S4d, Supporting Information, it is shown that with the decrease of percent of Au in the nanoalloys, the LSPR peak wavelengths keep moving toward lower wavelength regime. Hence, by incorporating other transition metals into the VAN film to form multialloyed nanopillar structures, one can expect to noticeably tailor the plasmonic resonance of the VAN system in much wider wavelength/frequency regions.

The dielectric permittivity of the BTO: (Au*‐*Co‐Pd) and BTO: (Au‐Pd) VAN films are plotted in Figure 4b and S6, Supporting Information. Owing to the anisotropic structure of VAN, angular‐dependent ellipsometry measurements were performed and fitted using a biaxial model to retrieve the in‐plane (IP) $\varepsilon_{∥}$ and out‐of‐plane (OOP) $\varepsilon_{⊥}$ permittivity values, respectively. Figure S5, Supporting Information, shows the measured and fitted parameters, Psi (Ψ) and Delta (Δ) over a wavelength range from 300 to 2000 nm. The real part permittivity of both films consisting of IP ($\varepsilon_{∥}^{\prime }$) and OOP ($\varepsilon_{⊥}^{\prime }$) components are plotted in Figure 4b. It can be seen that $\varepsilon_{∥}^{\prime }$ of both films are positive across the entire wavelength range (300–2000 nm), implying the dielectric characteristic of both VAN films along the IP direction.^[^ ^21^, ^22^, ^23^, ^36^, ^41^ ^]^ On the contrary, both $\varepsilon_{⊥}^{\prime }$ curves transition from positive to negative, showing the Type I hyperbolic metamaterials (HMM) ($\varepsilon_{∥}^{\prime }$ > 0, $\varepsilon_{⊥}^{\prime }$ < 0) behavior.^[^ ^17^, ^60^ ^]^ Hyperbolic dispersion enables high‐*k* wave propagation and enhances the photonic density of states. This enables applications like hyperlenses and optical sensing in the visible (Vis) and near‐infrared regions (NIR).^[^ ^60^, ^61^, ^62^ ^]^ The schematic inset in Figure 4b shows the hyperboloidal iso‐frequency topology surface of the BTO: (Au*‐*Co‐Pd) at 1500 nm wavelength regime. It is worth noting that the HMM transition wavelengths in the two hybrid films exhibit a significant blue shift from 1650 nm for BTO: (Au‐Pd) to 1120 nm for BTO: (Au*‐*Co‐Pd), indicating the higher free electron concentration in the Au*‐*Co‐Pd alloyed film,^[^ ^17^, ^21^, ^22^, ^23^, ^36^, ^63^ ^]^ which is also consistent with the LSPR shift observed in Figure 4a. The imaginary permittivity curves of both films are plotted in Figure S6, Supporting Information. It is noted that the OP $\varepsilon_{⊥}\prime \prime$ component of the BTO: (Au*‐*Co‐Pd) film shows overall higher value in the Vis‐NIR wavelength region, implying the high loss feature. The refractive index (*n*) and the extinction coefficient (*k*) of two films are further retrieved and plotted in Figure S7, Supporting Information. Both IP $n_{∥}$ and OOP $n_{⊥}$ results show the plasmonic absorption signature peaks in both hybrid films, and the OOP $k_{⊥}$ curves exhibit the higher loss feature in the trimetallic hybrid film.

**Figure 5:**
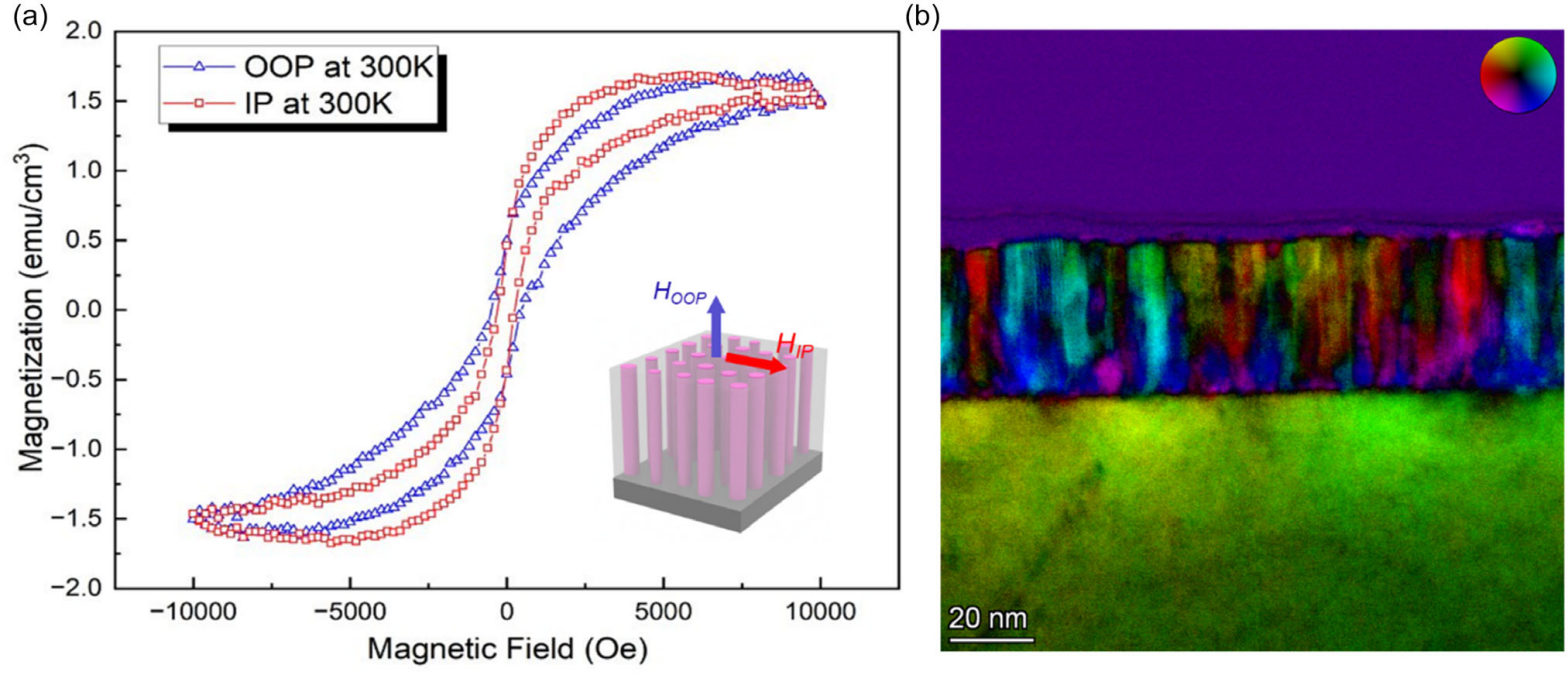
a) Magnetism hysteresis (*M‐H*) loops of BTO: (Au*‐*Co‐Pd) nanocomposite film along OOP and IP directions measured at 300 K. Inset shows the 3D schematic of the measurement. b) Reconstructed magnetic field map of BTO: (Au*‐*Co‐Pd) nanocomposite film based on the DPC‐STEM experiment.

The BTO: (Au*‐*Co‐Pd) hybrid film is expected to exhibit magnetic response due to the incorporated Co ferromagnetic phase. **Figure** **5**a shows the magnetization versus magnetic field (*M‐H*) hysteresis loops of the film measured at room temperature (T = 300 K). Both IP and OOP exhibit minimal magnetic saturation (*M*_s_) values due to the very small concentration of Co (based on the EDS analysis). Nanopillar geometry, particularly density and alignment, plays a key role in tuning magnetic properties in VAN films because of shape anisotropy, interpillar magnetic coupling, and variations in magnetic volume, all of which influence saturation magnetization and coercivity, especially in the OOP direction.^[^ ^64^ ^]^ The magnetic characteristic of the BTO: (Au*‐*Co‐Pd) hybrid film is further clarified by differential phase contrast (DPC)‐STEM imaging. The DPC‐STEM images acquired by a four‐quadrant annular detector are shown in Figure S8a–d, Supporting Information. The different phase contrast in the DPC‐STEM images is attributed to the deflection of the electron beam as it passes through the magnetic specimen in TEM.^[^ ^65^ ^]^ The reconstructed magnetic field map (Figure 5b) shows different oriented magnetic domain structures of the BTO: (Au*‐*Co‐Pd) hybrid film. The colored substrate area is attributed to the diffraction contrast effect caused by the thickness gradient contour at the mechanical polished TEM sample edge. Future more careful calibration work is needed at different sample tilting angles to minimize the diffraction contrast and achieve quantitative results in DPC‐STEM imaging.^[^ ^66^, ^67^ ^]^

This work demonstrates a simplified oxide‐metal strips deposition method of fabricating oxide: multialloy nanocomposite thin films with tailorable multifunctionalities. This one‐step simple deposition approach can further expand the materials selection to the synthesis of diverse transitional metal oxides (nitrides, carbides, and so on) coupled with various metallic phases toward versatile properties and functionalities applications. Future efforts could more focus on the precise control over the wide range of architecture of the oxide‐alloy VANs, that is, ordered distribution of secondary metallic phases as nanopillars in the primary oxide matrix. In addition, the permittivity tuning and dielectric loss mechanisms remain unexplored in many material systems such as oxides and transition metal dichalcogenides (TMDs). The recent studies demonstrate that the incorporation of rare earth elements and annealing‐induced lattice strains can effectively modulate the electronic structures in TMD absorbing materials.^[^ ^68^, ^69^ ^]^ Moreover, the dynamical properties tuning such as LSPR shifts and permittivity tuning under external stimuli (e.g., electrical bias, heating, and so on) can further induce exotic physical phenomena and expand the application range of the hybrid material systems.^[^ ^70^, ^71^ ^]^ Broadening the optical properties tuning range from UV–Vis‐NIR to short‐ and mid‐wavelength infrared regimes can further open up more intriguing topics for IR light photodetectors and absorbing materials that have great potential applications in 5G and quantum computing technologies.^[^ ^72^, ^73^, ^74^ ^]^

## 3 Conclusion

We have successfully fabricated the self‐assembled BTO: (Au*‐*Co‐Pd) and BTO: (Au‐Pd) VAN thin films via a simplified oxide‐metal strips deposition method in PLD. The XRD, STEM, and FFT analysis demonstrate the overall epitaxial growth of the film with some local nonuniformity and strain‐induced defects. The EDS analytical results confirm the elemental segregation for Au, Pd, and Co within the nanopillar structures due to the different surface energies of metals. The experimental transmittance spectra, simulated EFMs, and fitted permittivity curves indicate the noticeable LSPR peak shift due to the free electron density change within the Au*‐*Co‐Pd and Au‐Pd alloy nanopillars. Owing to the unique structure anisotropy, the VAN films exhibit hyperbolic dispersion feature in the Vis‐NIR wavelength region. The BTO: (Au*‐*Co‐Pd) VAN film shows weak ferromagnetic response due to the minor amount of Co in the hybrid film. The successful incorporation of catalytic Pd into the VAN system has opened potential applications of VAN films in the fields of catalysis and energy storage applications. Furthermore, the simplified oxide‐metal strips deposition used in this work can be applied to many other materials systems for the direct integration of diverse transition metals into VAN framework toward versatile device applications.

## 4 Experimental Section

4.1

4.1.1

##### Thin Film Growth

The BaTiO_3_ (BTO) target was prepared using a conventional solid‐state method. All the Au, Co, and Pd pure metals were cut as narrow strips and stuck on the BTO target surface for deposition. A PLD system with a KrF excimer laser (Lambda Physik Compex Pro 205, *λ* = 248 nm) was used for all film growth. The deposition parameters were set with a laser energy density of ≈2.0 J cm^−2^, a frequency of 2 Hz, and an oxygen partial pressure of 100 mTorr. The laser was focused on the target surface at a 45° angle of incidence, with a deposition temperature of 600 °C. The target‐substrate distance was ≈4.5 cm. After deposition, the films were cooled to room temperature at a rate of 10 °C min^−1^ under 100 mTorr O_2_ atmosphere.

##### Microstructure Characterization

XRD (Panalytical X’Pert X‐ray Diffractometer) with a Cu Kα1 radiation source with *λ* = 0.154 nm was employed to assess material crystallinity. An RSM was performed using a high‐resolution four‐circle X‐ray diffractometer (Smartlab, Rigaku Inc.). A Thermo Fisher Scientific (FEI) Talos F200X TEM running at 200 kV was used for sample imaging. HRSTEM imaging was conducted using a modified FEI Titan microscope with aberration‐correction technology, which enhances imaging resolution operating at a voltage of 300 kV. EDS analysis was conducted with a high‐resolution FEI Titan G2 80‐200 STEM equipped with ChemiSTEM technology, which aids in chemical analysis and mapping of thin films at high spatial resolution. Cross‐sectional TEM samples were prepared using a conventional method, including manual grinding, polishing, dimpling, and ion milling (PIPS 695, Gatan Inc.). Additionally, 3D electron tomography was performed using a Fischione Model 2045 Motorized Dual‐Axis tomography holder, followed by Inspect 3D and Tomviz for data reconstruction and visualization, respectively. The DPC imaging was performed on FEI Talox F200X equipped with four segmented STEM detectors at low‐magnification STEM mode to minimize the influence of the objective lens’ magnetic field on the specimen.

##### Optical Property Measurements and Simulation

Normal incidence depolarized transmittance (T%) spectra were collected using a UV–vis‐NIR absorption spectrophotometer (Perkin Elmer Lambda 1050) in the range of 200–1000 nm. Ellipsometry measurements were performed with a spectroscopic ellipsometer (J.A. Woollam RC_2_) at various angles (55^0^, 65^0^, and 75^0^) to obtain ellipsometry parameters psi (Ψ) and delta (Δ), which were used to derive the dielectric permittivity values of the films. These are related to the reflection coefficients for p‐polarized (r_p_) and s‐polarized (r_s_) light by the equation r_p_/r_s_ = tan (Ψ)⋅e^iΔ^. The dielectric permittivity of the films was derived by fitting these ellipsometry parameters using the Lorentz oscillators in the CompleteEASE software. All the final fitted results have mean square error less than 3.0.

COMSOL Multiphysics Wave Optics Module, the electromagnetic waves and frequency domain (*ewfd*), was applied to simulate the optical properties of hybrid films. Optical constants for materials such as Au, Co, Pd, and BaTiO_3_ were directly taken from the COMSOL software database. The refractive index (*n*) and extinction coefficient (*k*) of Au_0.9_Co_0.1_, Au_0.8_Co_0.05_Pd_0.15_, and Au_0.7_Pd_0.3_ are calculated using the MG model based on the EMA theory.^[^ ^55^ ^]^ The simulated geometry parameters (i.e., film thickness, nanopillar diameter, average distance, and so on) were retrieved from the cross‐sectional STEM images of those hybrid films. Normal incidence depolarized electromagnetic fields with ports placed on the top and bottom of the model were used to simulate the optical characteristics of the hybrid films.

##### Magnetic Property Measurement

The magnetic properties of the sample were investigated using a Vibrating Sample Magnetometer in a Magnetic Property Measurement System (MPMS‐3, from Quantum Design Inc.). Both IP and OOP magnetization were measured at 300 K by applying a magnetic field of 1 T, parallel and perpendicular to the sample plane. The diamagnetic substrate contribution was subtracted before plotting the *M‐H* hysteresis loops to ensure accurate results.

## Supporting Information

Supporting Information is available from the Wiley Online Library or from the author.

## Conflict of Interest

The authors declare no conflict of interest.

## Supporting information

Supplementary Material

## Data Availability

The data that support the findings of this study are available in the supplementary material of this article.

## References

[1] C. H. Ahn , K. M. Rabe , J.‐M. Triscone , Science 1979 2004, 303, 488. 10.1126/science.1092508 14739450

[2] H. N. Lee , H. M. Christen , M. F. Chisholm , C. M. Rouleau , D. H. Lowndes , Nature 2005, 433, 395. 15674286 10.1038/nature03261

[3] A. I. Buzdin , Rev. Mod. Phys. 2005, 77, 935.

[4] R. Yan , G. Khalsa , S. Vishwanath , Y. Han , J. Wright , S. Rouvimov , D. S. Katzer , N. Nepal , B. P. Downey , D. A. Muller , Nature 2018, 555, 183. 29516996 10.1038/nature25768

[5] S. Das , Y. L. Tang , Z. Hong , M. A. P. Gonçalves , M. R. McCarter , C. Klewe , K. X. Nguyen , F. Gómez‐Ortiz , P. Shafer , E. Arenholz , Nature 2019, 568, 368. 30996320 10.1038/s41586-019-1092-8

[6] A. R. Damodaran , J. D. Clarkson , Z. Hong , H. Liu , A. K. Yadav , C. T. Nelson , S.‐L. Hsu , M. R. McCarter , K.‐D. Park , V. Kravtsov , Nat. Mater. 2017, 16, 1003. 28783161 10.1038/nmat4951

[7] J. L. MacManus‐Driscoll , P. Zerrer , H. Wang , H. Yang , J. Yoon , A. Fouchet , R. Yu , M. G. Blamire , Q. Jia , Nat. Mater. 2008, 7, 314. 18311144 10.1038/nmat2124

[8] A. Chen , Z. Bi , Q. Jia , J. L. MacManus‐Driscoll , H. Wang , Acta Mater. 2013, 61, 2783.

[9] W. Zhang , A. Chen , Z. Bi , Q. Jia , J. L. MacManus‐Driscoll , H. Wang , Curr. Opin. Solid State Mater. Sci. 2014, 18, 6.

[10] S. Misra , H. Wang , Mater. Horiz. 2021, 8, 869. 34821319 10.1039/d0mh01111h

[11] S. M. Yang , S. Lee , J. Jian , W. Zhang , P. Lu , Q. Jia , H. Wang , T. Won Noh , S. V. Kalinin , J. L. MacManus‐Driscoll , Nat. Commun. 2015, 6, 8588. 26446866 10.1038/ncomms9588 PMC4633963

[12] A. Chen , J.‐M. Hu , P. Lu , T. Yang , W. Zhang , L. Li , T. Ahmed , E. Enriquez , M. Weigand , Q. Su , Sci. Adv. 2016, 2, e1600245. 27386578 10.1126/sciadv.1600245 PMC4928986

[13] H. Liu , H. Wu , K. P. Ong , T. Yang , P. Yang , P. K. Das , X. Chi , Y. Zhang , C. Diao , W. K. A. Wong , Science 1979 2020, 369, 292. 10.1126/science.abb3209 32675370

[14] H. Zheng , J. Wang , S. E. Lofland , Z. Ma , L. Mohaddes‐Ardabili , T. Zhao , L. Salamanca‐Riba , S. R. Shinde , S. B. Ogale , F. Bai , Science 1979 2004, 303, 661. 10.1126/science.1094207 14752158

[15] A. Kumar , S. Ning , T. Su , E. Cho , J. M. LeBeau , C. A. Ross , Adv. Electron. Mater. 2022, 8, 2200036.

[16] X. Gao , L. Li , J. Jian , H. Wang , M. Fan , J. Huang , X. Wang , H. Wang , ACS Appl. Nano Mater. 2018, 1, 2509.

[17] D. Zhang , H. Wang , Adv. Photonics Res. 2021, 2, 2000174.

[18] J. L. MacManus‐Driscoll , S. R. Foltyn , Q. X. Jia , H. Wang , A. Serquis , L. Civale , B. Maiorov , M. E. Hawley , M. P. Maley , D. E. Peterson , Nat. Mater. 2004, 3, 439. 15170180 10.1038/nmat1156

[19] Q. Su , W. Zhang , P. Lu , S. Fang , F. Khatkhatay , J. Jian , L. Li , F. Chen , X. Zhang , J. L. MacManus‐Driscoll , ACS Appl. Mater. Interfaces 2016, 8, 20283. 27438729 10.1021/acsami.6b05999

[20] L. Li , L. Sun , J. S. Gomez‐Diaz , N. L. Hogan , P. Lu , F. Khatkhatay , W. Zhang , J. Jian , J. Huang , Q. Su , Nano Lett. 2016, 16, 3936. 27186652 10.1021/acs.nanolett.6b01575

[21] D. Zhang , S. Misra , L. Li , X. Wang , J. Jian , P. Lu , X. Gao , X. Sun , Z. Qi , M. Kalaswad , Adv. Opt. Mater. 2020, 8, 1901359.

[22] D. Zhang , P. Lu , S. Misra , A. Wissel , Z. He , Z. Qi , X. Gao , X. Sun , J. Liu , J. Lu , Adv. Opt. Mater. 2021, 9, 2001154.

[23] S. Misra , L. Li , D. Zhang , J. Jian , Z. Qi , M. Fan , H. Chen , X. Zhang , H. Wang , Adv. Mater. 2019, 31, 1806529. 10.1002/adma.201806529 30575142

[24] J. Huang , X. Wang , N. L. Hogan , S. Wu , P. Lu , Z. Fan , Y. Dai , B. Zeng , R. Starko‐Bowes , J. Jian , Adv. Sci. 2018, 5, 1800416. 10.1002/advs.201800416 PMC6051386 30027062

[25] X. Wang , J. Jian , S. Diaz‐Amaya , C. E. Kumah , P. Lu , J. Huang , D. G. Lim , V. G. Pol , J. P. Youngblood , A. Boltasseva , Nanoscale Adv. 2019, 1, 1045. 36133204 10.1039/c8na00306h PMC9473282

[26] X. Wang , J. Jian , Z. Zhou , C. Fan , Y. Dai , L. Li , J. Huang , J. Sun , A. Donohue , P. Bermel , Adv. Opt. Mater. 2019, 7, 1801180.

[27] X. Wang , H. Wang , Nanoscale 2020, 12, 20564. 33090168 10.1039/d0nr06316a

[28] X. Wang , H. Wang , J. Jian , B. X. Rutherford , X. Gao , X. Xu , X. Zhang , H. Wang , Nano Lett. 2020, 20, 6614. 32787175 10.1021/acs.nanolett.0c02440

[29] J. Song , D. Zhang , P. Lu , H. Wang , X. Xu , M. L. Meyerson , S. G. Rosenberg , J. Deitz , J. Liu , X. Wang , Mater. Today Nano 2023, 22, 100316.

[30] J. Song , D. Zhang , M. Moceri , H. Dou , X. Zhang , H. Wang , Adv. Mater. Interfaces 2024, 11, 2400132.

[31] J. Huang , X. Wang , X. L. Phuah , P. Lu , Z. Qi , H. Wang , Mater. Today Nano 2019, 8, 100052.

[32] J. Huang , L. Li , P. Lu , Z. Qi , X. Sun , X. Zhang , H. Wang , Nanoscale 2017, 9, 7970. 28574068 10.1039/c7nr01122a

[33] B. Zhang , J. Huang , J. Jian , B. X. Rutherford , L. Li , S. Misra , X. Sun , H. Wang , Nanoscale Adv. 2019, 1, 4450. 36134413 10.1039/c9na00438f PMC9417828

[34] J. Huang , Z. Qi , L. Li , H. Wang , S. Xue , B. Zhang , X. Zhang , H. Wang , Nanoscale 2018, 10, 17182. 30191234 10.1039/c8nr05532g

[35] B. Zhang , J. Huang , B. X. Rutherford , P. Lu , S. Misra , M. Kalaswad , Z. He , X. Gao , X. Sun , L. Li , Mater. Today Nano 2020, 11, 100083.

[36] D. Zhang , S. Misra , J. Jian , P. Lu , L. Li , A. Wissel , X. Zhang , H. Wang , ACS Appl. Mater. Interfaces 2021, 13, 5390. 33464819 10.1021/acsami.0c19108

[37] R. L. Paldi , X. Wang , X. Sun , Z. He , Z. Qi , X. Zhang , H. Wang , Nano Lett. 2020, 20, 3778. 32330053 10.1021/acs.nanolett.0c00790

[38] J. Huang , Y. Fang , P. Lu , J. Lu , H. Wang , Nano Res. 2024, 17, 8226.

[39] R. Wu , D. Zhang , T. Maity , P. Lu , J. Yang , X. Gao , S. Zhao , X. Wei , H. Zeng , A. Kursumovic , Nat. Electron. 2021, 4, 333.

[40] M. Hennes , X. Weng , E. Fonda , B. Gallas , G. Patriarche , D. Demaille , Y. Zheng , F. Vidal , Phys. Rev. Mater. 2019, 3, 035002.

[41] J. Huang , X. L. Phuah , L. M. McClintock , P. Padmanabhan , K. S. N. Vikrant , H. Wang , D. Zhang , H. Wang , P. Lu , X. Gao , Mater. Today 2021, 51, 39.

[42] B. X. Rutherford , H. Dou , B. Zhang , Z. He , J. P. Barnard , R. L. Paldi , H. Wang , Nanomaterials 2022, 12, 3460. 36234589 10.3390/nano12193460 PMC9565752

[43] J. Huang , B. Zhang , D. Hermawan , A. Sanjuan , B. K. Tsai , J. Huang , R. E. Garćıa , H. Wang , Adv. Funct. Mater. 2025, 35, 2500741.

[44] J. Song , D. Zhang , P. Lu , Y. Zhang , H. Wang , H. Dou , X. Xu , J. Deitz , X. Zhang , H. Wang , ACS Appl. Mater. Interfaces 2023, 15, 37810. 37493477 10.1021/acsami.3c06777

[45] B. X. Rutherford , B. Zhang , M. Kalaswad , Z. He , D. Zhang , X. Wang , J. Liu , H. Wang , ACS Appl. Nano Mater. 2022, 5, 6297.

[46] X. Wang , J. Jian , H. Wang , J. Liu , Y. Pachaury , P. Lu , B. X. Rutherford , X. Gao , X. Xu , A. El‐Azab , Small 2021, 17, 2007222. 10.1002/smll.202007222 33448118

[47] D. Zhang , K. J. Harmon , M. J. Zachman , P. Lu , D. Kim , Z. Zhang , N. Cucciniello , R. Markland , K. W. Ssennyimba , H. Zhou , InfoMat 2024, 6, e12561.

[48] B. X. Rutherford , D. Zhang , L. Quigley , J. P. Barnard , B. Yang , J. Lu , S. Kunwar , H. Dou , J. Shen , A. Chen , Small Science 2023, 3, 2300049. 40213517 10.1002/smsc.202300049 PMC11935946

[49] R. Yuan , A. Kumar , S. Zhuang , N. Cucciniello , T. Lu , D. Xue , A. Penn , A. R. Mazza , Q. Jia , Y. Liu , Nano Lett 2023, 23, 4807. 37224193 10.1021/acs.nanolett.3c00277

[50] R. Chinchilla , C. Najera , Chem. Rev. 2014, 114, 1783. 23789922 10.1021/cr400133p

[51] L. Yin , J. Liebscher , Chem. Rev. 2007, 107, 133. 17212474 10.1021/cr0505674

[52] J. W. Arblaster , Platin. Met. Rev. 2012, 56, 181. 10.1595/147106712x629761 PMC3608409 23555153

[53] L. Vitos , A. V. Ruban , H. L. Skriver , J. Kollár , Surf. Sci. 1998, 411, 186.

[54] W. Wang , Q. Zhou , Y. Dong , E. S. Tok , Y.‐C. Yeo , Appl. Phys. Lett. 2015, 106, 232106.

[55] S. Pereira , M. R. Correia , E. Pereira , K. P. O’Donnell , E. Alves , A. D. Sequeira , N. Franco , I. M. Watson , C. J. Deatcher , Appl. Phys. Lett. 2002, 80, 3913.

[56] H. Stanchu , A. V. Kuchuk , Y. I. Mazur , J. Margetis , J. Tolle , J. Richter , S.‐Q. Yu , G. J. Salamo , Semicond. Sci. Technol. 2020, 35, 075009.

[57] W. L. Barnes , A. Dereux , T. W. Ebbesen , Nature 2003, 424, 824. 12917696 10.1038/nature01937

[58] G. A. Niklasson , C. G. Granqvist , O. Hunderi , Appl. Opt. 1981, 20, 26. 20309062 10.1364/AO.20.000026

[59] J. I. Gittleman , B. Abeles , Phys. Rev. B 1977, 15, 3273.

[60] C. L. Cortes , W. Newman , S. Molesky , Z. Jacob , J. Optics 2012, 14, 063001.

[61] Z. Jacob , L. V. Alekseyev , E. Narimanov , Opt. Express 2006, 14, 8247. 19529199 10.1364/oe.14.008247

[62] S. Kawata , Y. Inouye , P. Verma , Nat. Photon. 2009, 3, 388.

[63] S. Misra , D. Zhang , Z. Qi , D. Li , J. Lu , H.‐T. Chen , H. Wang , Cryst. Growth Des. 2020, 20, 6101.

[64] Y. Zhang , J. Song , P. Lu , J. Deitz , D. Zhang , H. Dou , J. Shen , Z. Hu , X. Zhang , H. Wang , Adv. Mater. Interfaces 2023, 10, 2300150.

[65] D. Zhang , X. Gao , J. Lu , P. Lu , J. Deitz , J. Shen , H. Dou , Z. He , Z. Shang , C. A. Wade , Nano Res. 2023, 16, 1465.

[66] N. Shibata , S. D. Findlay , Y. Kohno , H. Sawada , Y. Kondo , Y. Ikuhara , Nat. Phys. 2012, 8, 611.

[67] A. Nakamura , Y. Kohno , H. Sasaki , N. Shibata , Microsc. Microanal. 2017, 23, 1412.

[68] J. Wen , S. Hui , Q. Chang , G. Chen , L. Zhang , X. Fan , K. Tao , H. Wu , Adv. Funct. Mater. 2024, 34, 2410447.

[69] J. Wen , Y. Liu , S. Hui , L. Deng , L. Zhang , X. Fan , Q. Chen , X. Liu , X. Li , N. Yan , Matter 2025, 8, 102151.

[70] J. Wu , F. Yan , J. Zhao , L. Qian , T. Cheng , J. Su , L. Bi , Y. Huang , W. Wang , Z. Zhang , Adv. Funct. Mater. 2024, 34, 2411358.

[71] S. Misra , M. Kalaswad , D. Zhang , H. Wang , Mater. Res. Lett. 2020, 8, 321.

[72] J. Zha , M. Luo , M. Ye , T. Ahmed , X. Yu , D. Lien , Q. He , D. Lei , J. C. Ho , J. Bullock , Adv. Funct. Mater. 2022, 32, 2111970.

[73] M. Qin , L. Zhang , H. Wu , Adv. Sci. 2022, 9, 2105553. 10.1002/advs.202105553 PMC8981909 35128836

[74] A. Rogalski , J. Appl. Phys. 2003, 93, 4355.

