# Supplementary material for: Multialloy Au‐Co‐Pd Nanopillars‐in‐Oxide Hybrid Metamaterials with Tunable Optical and Magnetic Properties
Source: Small Sci. 2025 Nov 10;5(12):e202500455. doi: 10.1002/smsc.202500455 (PMC12697833; doi:10.1002/smsc.202500455)
Supplement: Supplementary file 1 — Supplementary Material [file SMSC-5-e202500455-s001.zip › smsc202500455-sup-0001-SuppData-S1.pdf]

Supporting Information

Multi-alloy Au-Co-Pd Nanopillars-in-Oxide Hybrid Metamaterials with Tunable Optical and Magnetic Properties

Vasundhara Acharya[1], Juanjuan Lu[2], Jiawei Song[2], Ping Lu[3], Alessandro R. Mazza[4,5], Jianan Shen[2], Zihao He[2], Juncheng Liu[2], Hongyi Dou[2], Yizhi Zhang[2], Zhongxia Shang[2], Aiping Chen[4], Haiyan Wang[2]*, Di Zhang[1]*

[1] Department of Materials Science and Engineering, University of Texas at Arlington, Arlington, 76019, USA
[2] School of Materials Engineering, Purdue University, West Lafayette, IN 47907, USA
[3] Sandia National Laboratories, Albuquerque, NM 87185, USA
[4] Center for Integrated Nanotechnologies, Los Alamos National Laboratory, Los Alamos, NM 87545, USA
[5] Materials Science and Technology Division, Los Alamos National Laboratory, Los Alamos, NM 87545, USA

Corresponding authors:

Di Zhang - Department of Materials Science and Engineering, University of Texas at Arlington, Arlington, TX 76019, USA. di.zhang@uta.edu

Haiyan Wang - School of Materials Engineering, Purdue University, West Lafayette, IN 47907, USA. hwang00@purdue.edu

Microstructure of BTO: (Au-Co-Pd) film

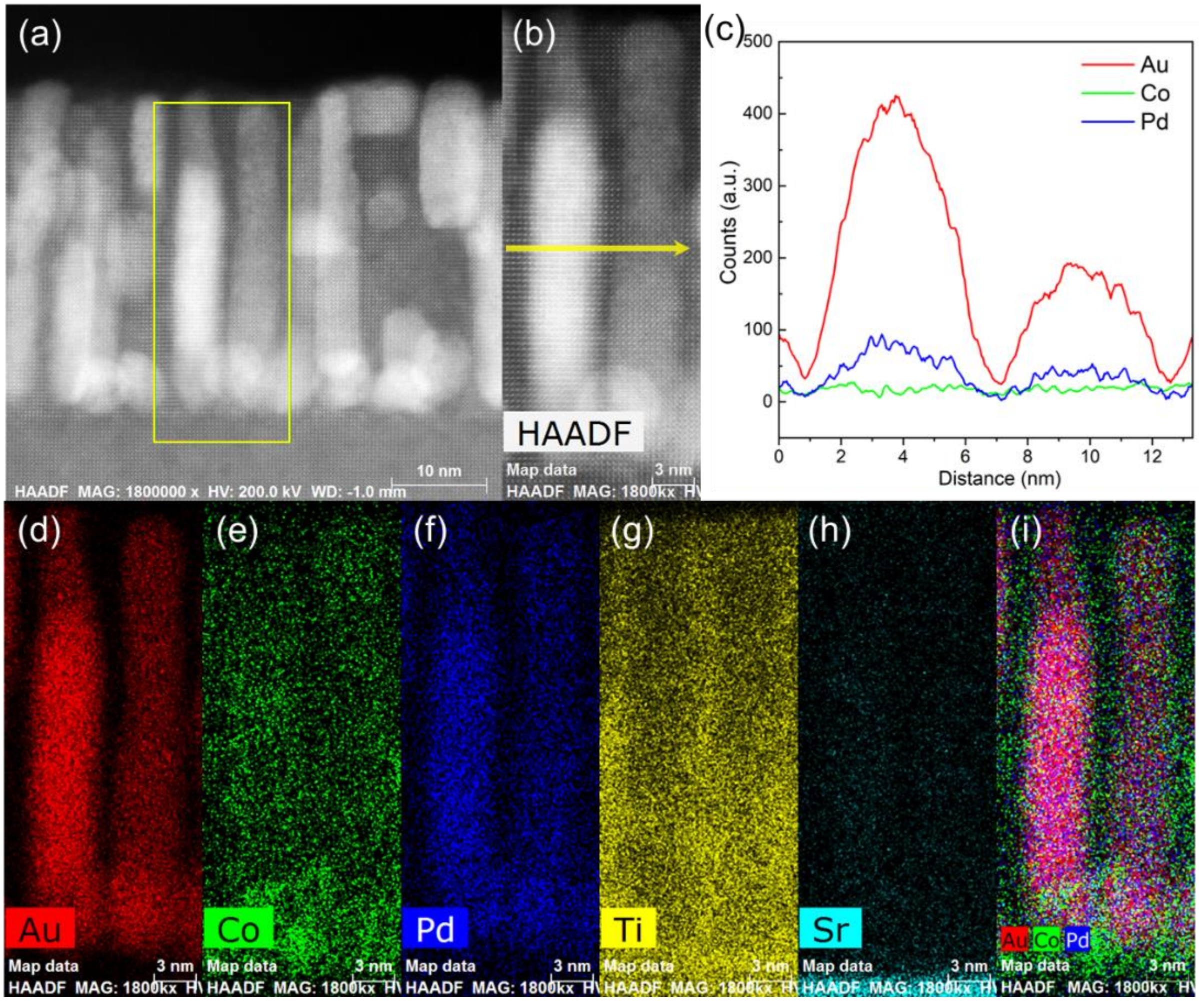

Figure S1. (a) Cross-sectional HAADF-STEM image of the BTO: (Au-Co-Pd) composite film. (b) Local HAADF image and corresponding EDS maps (d-i) for the same region. (c) EDS line profile across the nanopillars region denoted in (b).

Microstructure of BTO: (Au-Pd) film

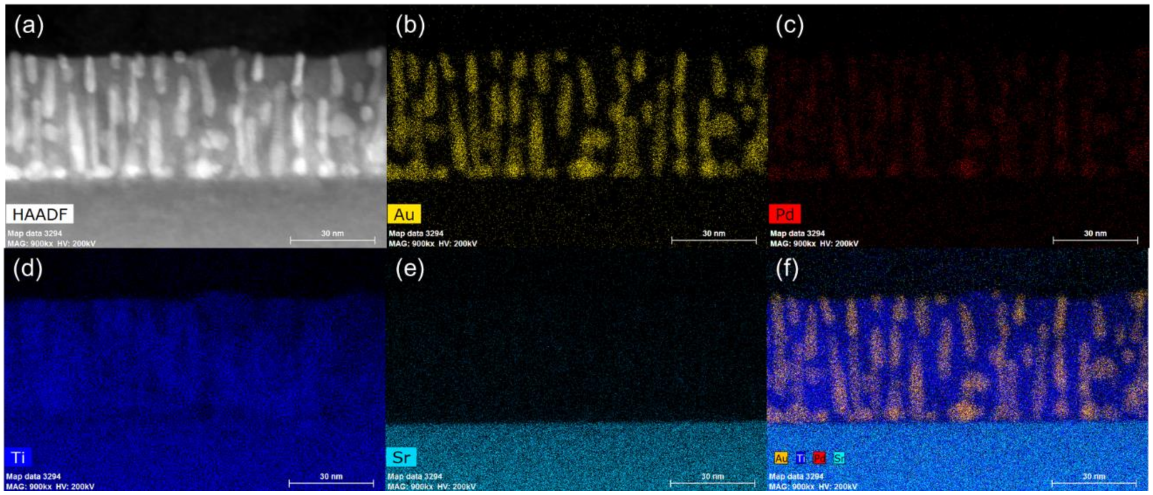

Figure S2. (a) HAADF image of BTO: (Au-Pd) composite film. Cross-section STEM-EDS map showing the elemental distribution of (b) Au, (c) Pd, (d) Ti, (e) Sr, and (f) BTO: (Au-Pd) composite film.

Refractive index (*n*) and extinction coefficient (*k*) of Au-Co, Au-Pd, and Au-Co-Pd alloys

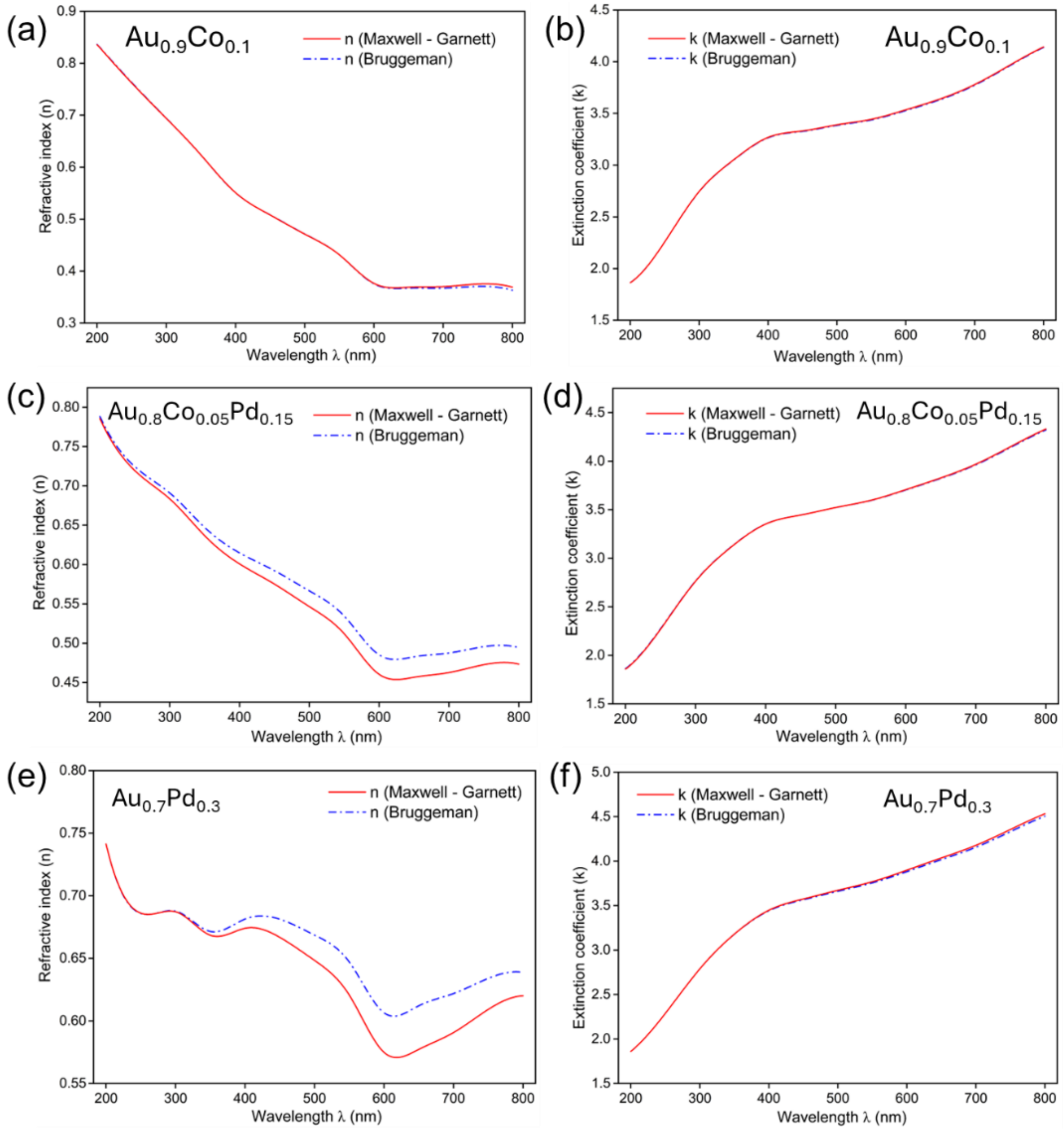

Figure S3. The calculated refractive index (*n*) and extinction coefficient (*k*) values of (a, b) $Au_{0.9}Co_{0.1}$, (c, d) $Au_{0.8}Co_{0.05}Pd_{0.15}$, and (e, f) $Au_{0.7}Pd_{0.3}$ alloys using the Maxwell-Garnett (MG) and Bruggeman (BG) models based on effective medium approximations (EMA).[1]

LSPR simulation at different normal incident light wavelengths

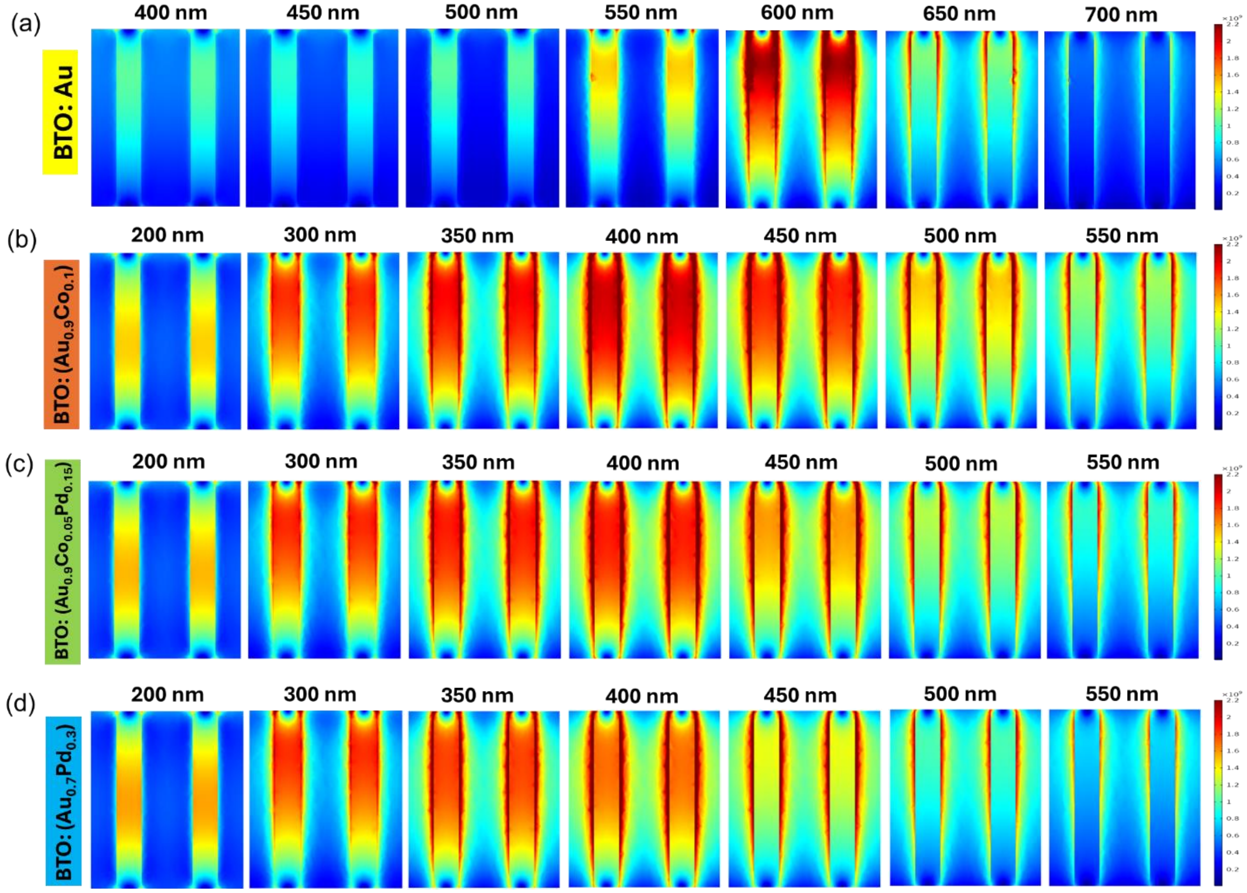

Figure S4. Cross-sectional electric field maps (EFMs) of the (a) BTO: Au, (b) BTO: $Au_{0.9}Co_{0.1}$, (c) BTO: $Au_{0.8}Co_{0.05}Pd_{0.15}$, and (d) BTO: $Au_{0.7}Pd_{0.3}$VAN films at different wavelengths of normal incident light illumination.

Experimental and fitted ellipsometric parameters Psi (Ψ) and Delta (Δ)

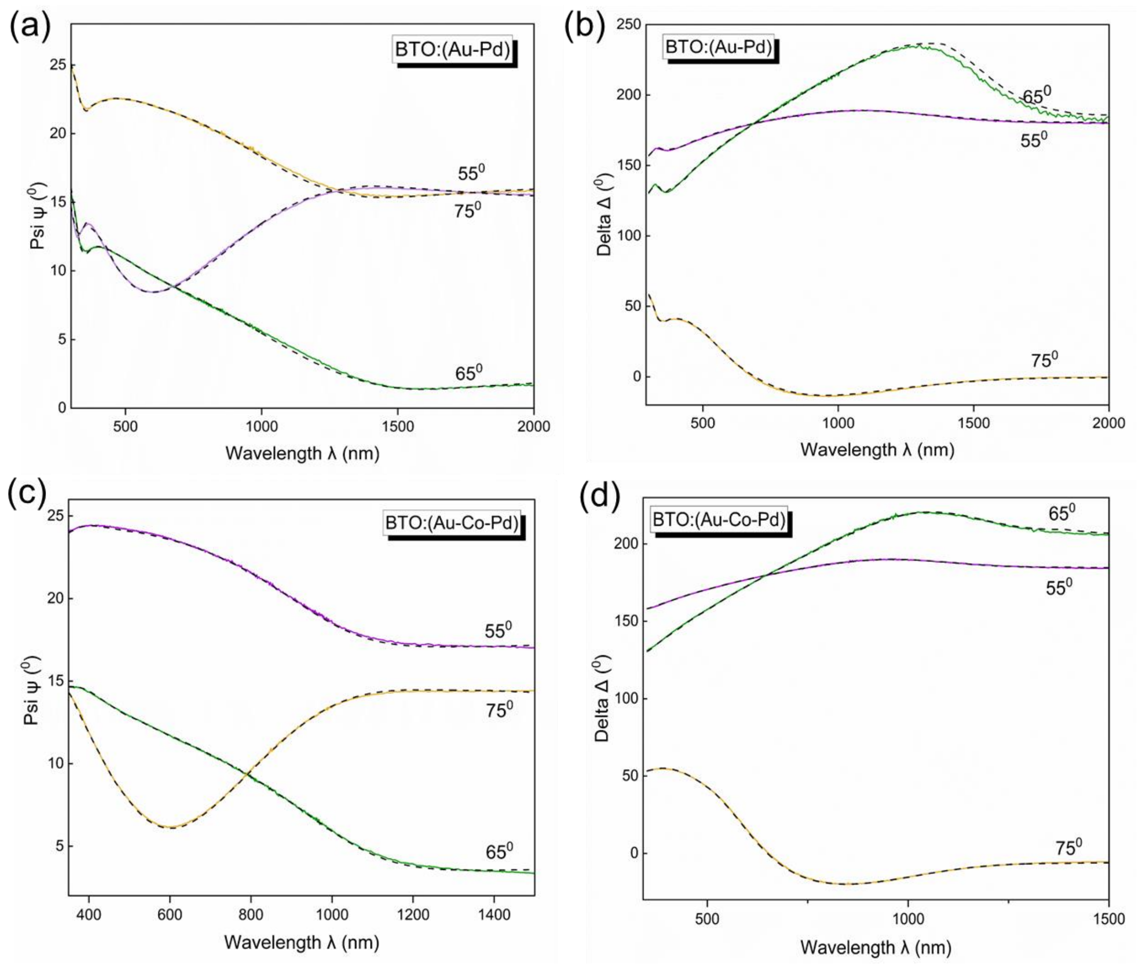

Figure S5. Experimental (dashed) and fitted (solid) ellipsometric parameters Psi (Ψ) and Delta (Δ) at incident angles of 55°, 65°, and 75° for the (a,b) BTO: (Au-Pd) and (c,d) BTO: (Au-Co-Pd) thin films.

The imaginary part dielectric permittivity

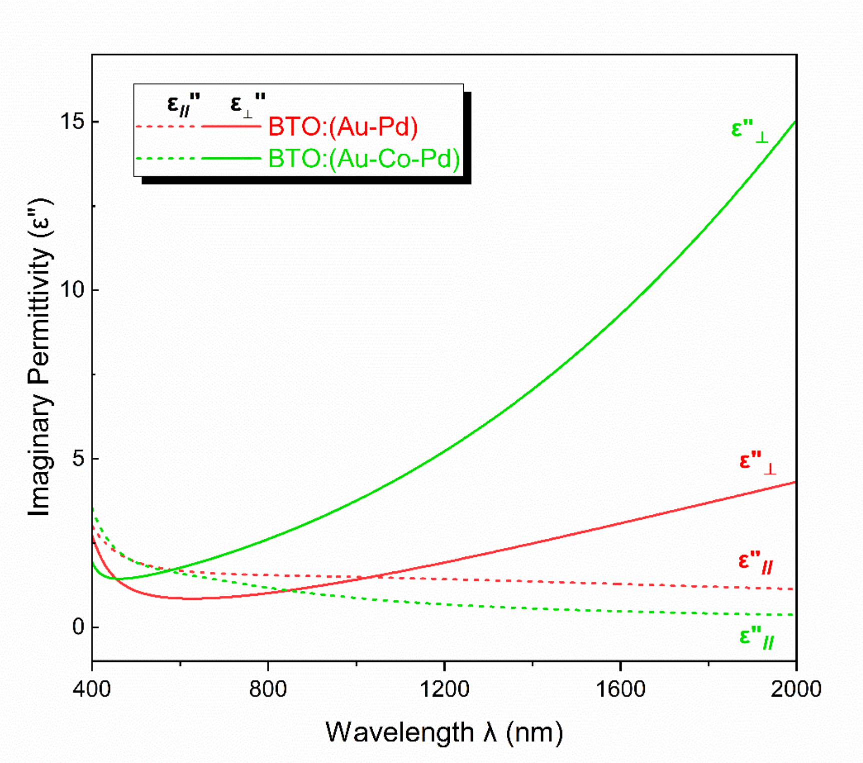

Figure S6. The imaginary part of the in-plane ($\varepsilon_{\parallel}''$) and out-of-plane ($\varepsilon_{\perp}''$) permittivity of the BTO: (Au-Pd) and BTO: (Au-Co-Pd) thin films.

Refractive index (*n*) and extinction coefficient (*k*)

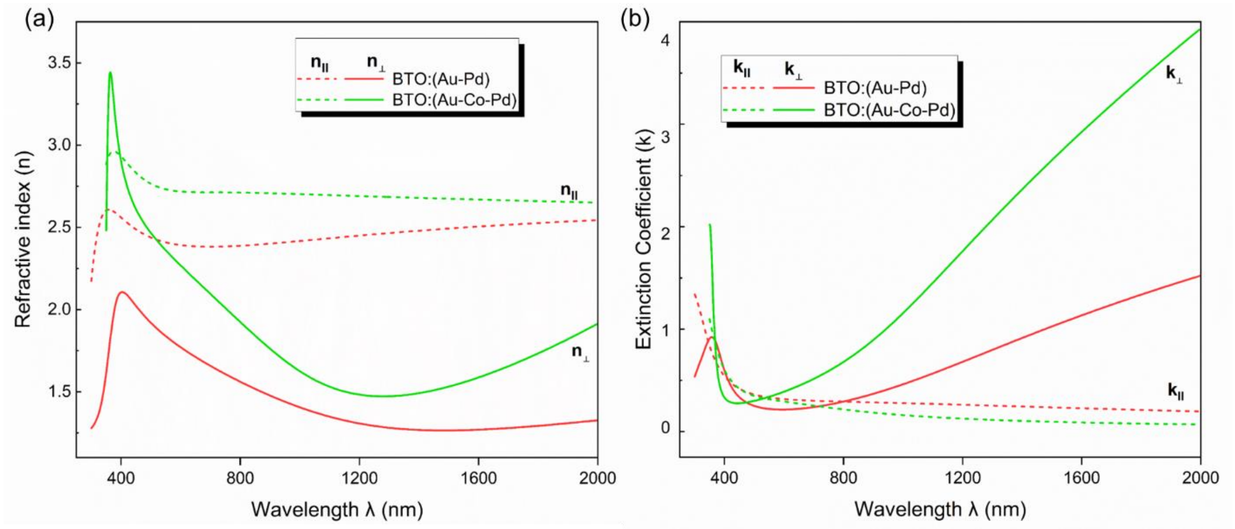

Figure S7. The in-plane (dashed line) and out-of-plane (solid line) components of (a) refractive index (*n*) and (b) extinction coefficient (*k*) of the BTO: (Au-Pd) and BTO: (Au-Co-Pd) thin films.

Differential phase contrast (DPC) Low-magnification STEM imaging

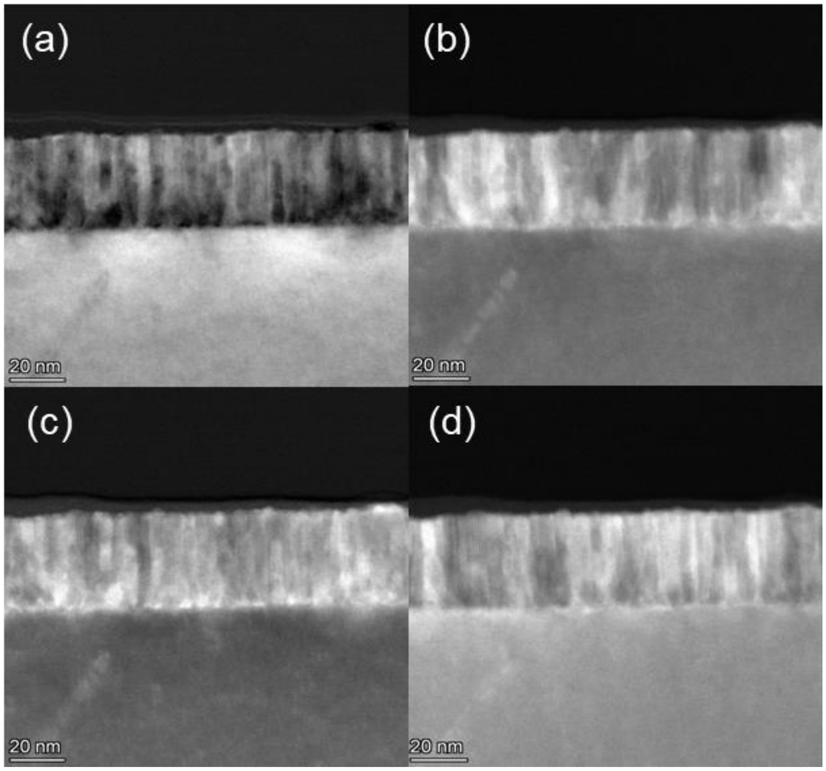

Figure S8. (a-d) The DPC-STEM images obtained by the four-quadrant annular detectors.

3D electron tomography movie

Movie S1. The video below shows the distribution of the Au-Co-Pd alloyed nanopillars in 3D space within the BTO: (Au-Co-Pd) nanocomposite thin film.

References:

[1] G. A. Niklasson, C. G. Granqvist, O. Hunderi, *Appl Opt* 1981, *20*, 26.
